# Fibrinogen to HDL-Cholesterol ratio as a predictor of mortality risk in patients with acute myocardial infarction

**DOI:** 10.1186/s12944-024-02071-7

**Published:** 2024-03-25

**Authors:** Congzhuo Jia, Wanying Wu, Huan Lu, Jin Liu, Shiqun Chen, Guoxiao Liang, Yang Zhou, Sijia Yu, Linfang Qiao, Jinming Chen, Ning Tan, Yong Liu, Jiyan Chen

**Affiliations:** 1 Department of Cardiology, Guangdong Cardiovascular Institute, Guangdong Provincial People’s Hospital, Guangdong Academy of Medical Sciences, Guangzhou, 510080 China; 2 Department of Guangdong Provincial Key Laboratory of Coronary Heart Disease Prevention, Guangdong Cardiovascular Institute, Guangdong Provincial People’s Hospital, Guangdong Academy of Medical Sciences, Guangzhou, 510080 China; 3 The Second School of Clinical Medicine, Southern Medical University, Guangzhou, 510515 China; 4 The School of Pharmacy, Guangdong Medical University, Dongguan, 523000 China; 5 Global Health Research Center, Guangdong Provincial People’s Hospital, Guangdong Academy of Medical Science, Guangzhou, 510100 China; 6 Department of Cardiology, Yangjiang People’s Hospital, Yangjiang, 529500 China

**Keywords:** Acute myocardial infarction, Fibrinogen, HDL cholesterol, Inflammation

## Abstract

**Background:**

Acute myocardial infarction (AMI) is characterized by inflammation, oxidative stress, and atherosclerosis, contributing to increased mortality risk. High-density lipoprotein (HDL) takes a crucial part in mitigating atherosclerosis and inflammation through its diverse functionalities. Conversely, fibrinogen is implicated in the development of atherosclerotic plaques. However, the mortality risk predictive capacity of fibrinogen to HDL-cholesterol ratio (FHR) in AMI patients remains unexplored. This research aimed to evaluate the effectiveness of FHR for mortality risk prediction in relation to AMI.

**Methods:**

A retrospective study involving 13,221 AMI patients from the Cardiorenal ImprovemeNt II cohort (NCT05050877) was conducted. Baseline FHR levels were used to categorize patients into quartiles. The assessment of survival disparities among various groups was conducted by employing Kaplan‒Meier diagram. Cox regression was performed for investigating the correlation between FHR and adverse clinical outcomes, while the Fine-Gray model was applied to evaluate the subdistribution hazard ratios for cardiovascular death.

**Results:**

Over a median follow-up of 4.66 years, 2309 patients experienced all-cause death, with 1007 deaths attributed to cardiovascular disease (CVD). The hazard ratio (HR) and its 95% confidence interval (CI) for cardiac and all-cause death among individuals in the top quartile of FHR were 2.70 (1.99–3.65) and 1.48 (1.26–1.75), respectively, in comparison to ones in the first quartile, after covariate adjustment. Restricted cubic spline analysis revealed that FHR was linearly correlated with all-cause mortality, irrespective of whether models were adjusted or unadjusted (all *P* for nonlinearity > 0.05).

**Conclusion:**

AMI patients with increased baseline FHR values had higher all-cause and cardiovascular mortality, regardless of established CVD risk factors. FHR holds promise as a valuable tool for evaluating mortality risk in AMI patients.

**Trial registration:**

The Cardiorenal ImprovemeNt II registry NCT05050877.

**Supplementary Information:**

The online version contains supplementary material available at 10.1186/s12944-024-02071-7.

## Introduction

Acute myocardial infarction (AMI), a significant thrombotic complication of atherosclerosis, remains a pivotal risk factor for morbidity and mortality. In-depth research underscores the involvement of lipoproteins, blood lipids, endothelial injury, and inflammation in the onset and advancement of atherosclerosis [1, 2]. Given the high prevalence of AMI, there is an imperative need for a better understanding of its causative factors to develop effective prognosis and treatment strategies [3].

Fibrinogen (FIB) has emerged as a key participant in atherosclerotic plaque development, influencing the function of endothelial cells [4, 5]. Besides, it is of critical significance in the coagulation cascade, profoundly influencing blood viscosity and platelet aggregation [6]. As an acute-phase protein, FIB exhibits enhanced biosynthesis, reaching plasma concentrations of several folds during inflammation [7]. Prior studies have proposed FIB as a prospective indicator for predicting the risk of cardiovascular outcomes [8]. Recent evidence has also indicated that FIB independently influences the progression of cardiovascular disease (CVD) and serves as a biomarker for inflammation and coagulation [9].

Conversely, high-density lipoprotein (HDL) cholesterol has been acknowledged as “good cholesterol” from early observational studies demonstrating its inverse relationship with the risk of cardiovascular disease [10, 11]. Recent advances have highlighted atheroprotective roles of HDL by reducing cell proliferation and inflammatory signaling pathways [12, 13]. HDL provides defense against endothelial injury and suppresses the expression of adhesion molecules during atherosclerosis development [14]. Furthermore, HDL plays a multifaceted role in regulating the coagulation cascade, positively correlating with anticoagulant responses and neutralizing the procoagulant effects of anionic phospholipids [15]. Although HDL has been linked with potential atheroprotective properties, efforts on raising HDL cholesterol levels through pharmacological interventions have failed to translate into reduced cardiovascular disease risk [16]. Emerging findings from genetic studies have further revealed that mutations leading to lower plasma HDL cholesterol levels are not correlated with higher risk of ischemic heart disease [17]. Additionally, a recent mendelian randomization study indicated genetic mechanisms that elevating plasma HDL cholesterol levels do not appear to decrease the risk of MI [18].

Thus, despite the contrasting significance of FIB and HDL in relation to coagulation and inflammatory alterations, their combined role in mortality prediction among patients with AMI remains largely unexplored. To date, limited studies have explored the coexistence of elevated FIB values and decreased HDL that were associated with recurrent cerebral venous thrombosis (CVT) among those previously diagnosed with CVT [19]. Additionally, this combination was closely correlated with the onset of CVD among diabetes patients [20].

Therefore, the research sought to explore the feasibility of utilizing the fibrinogen to HDL-cholesterol ratio (FHR) as an indicative marker for predicting adverse clinical outcomes in AMI population. The primary goal of this analysis was to evaluate potential biomarkers for better risk stratification and patient management in patients with high-risk AMI.

## Methods

### Study design and participants

**Fig. 1:**
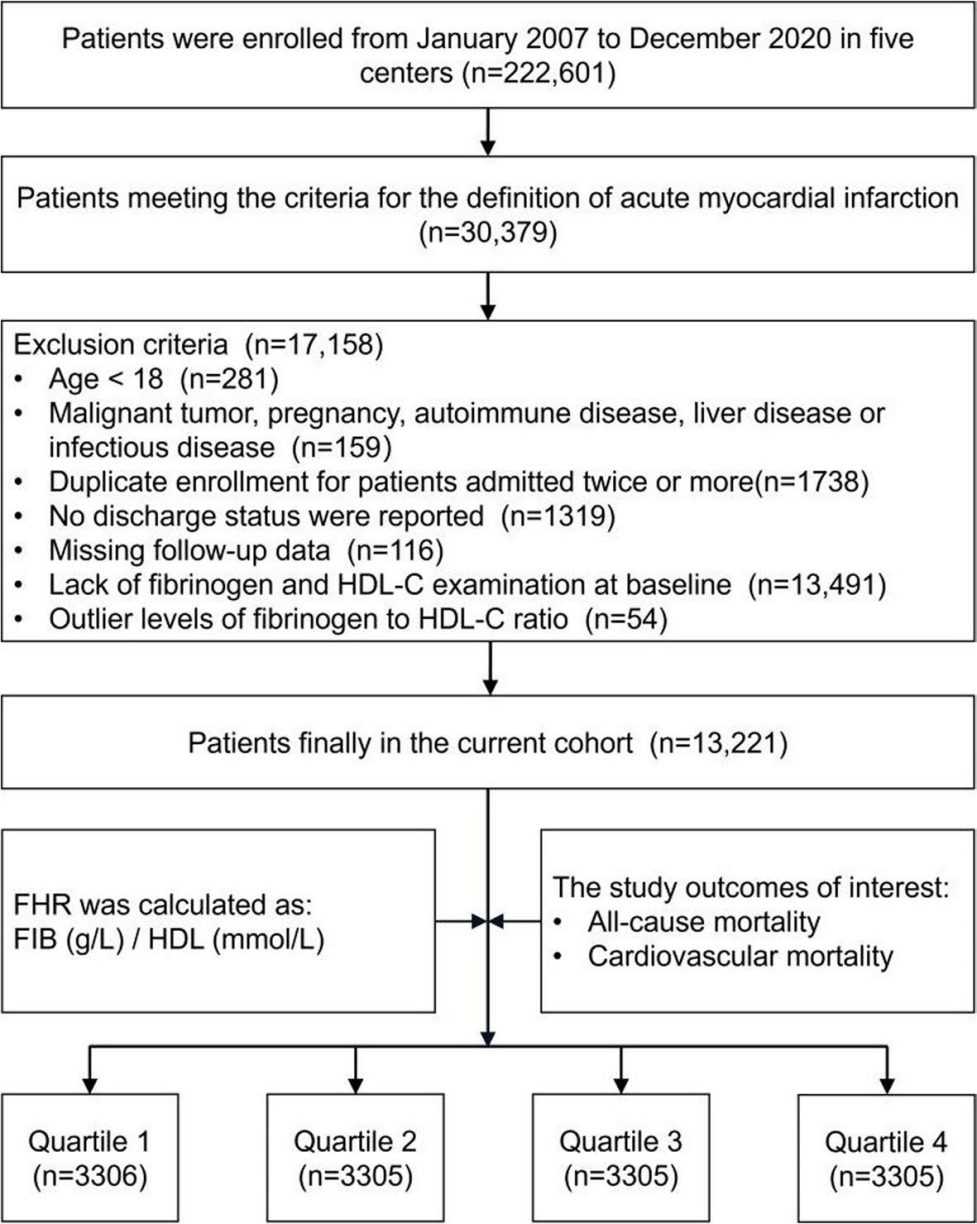
Study flowchart. *FHR* fibrinogen to HDL-cholesterol ratio

Data for this retrospective study were sourced from the multicenter Cardiorenal ImprovemeNt II (CIN-II, NCT05050877) cohort registry, covering the period from January 2007 to December 2020. The research was carried out across five central tertiary teaching hospitals located in different regions of southern China. The study included patients who met the following requirements for enrollment: 1) meeting the diagnostic criteria of AMI; 2) aged ≥ 18 years; 3) not presenting with any concomitant malignancy, pregnancy, autoimmune disease, liver disease or infectious disease; 4) including only the initial admission for patients admitted twice or more; 5) possessing complete baseline, discharge status, and follow-up data; and 6) not demonstrating an outlier baseline FHR (Fig. 1). The present study enrolled 13,221 patients for the final analysis. This study adhered to the Declaration of Helsinki and was endorsed by the Ethics Committee of Guangdong Provincial People’s Hospital.

### Data collection

This study collected data from the electronic clinical management system, which includes baseline information such as demographic features, coexisting conditions, laboratory tests, treatments during hospitalization, and discharge medications. Prior to blood sample extraction, participants were required to undergo a fasting period (> 8 h). Routine blood tests, fibrinogen, high-sensitivity troponin T (hs-TnT), total cholesterol (TC), triglyceride HDL and low-density lipoprotein (LDL) were tested by standard laboratory methods. Fibrinogen levels were assessed using the STA-R Evolution R System (Beijing Stago Diagnosis Trading Co., Ltd., Beijing, China) along with supplied reagents of the instrument (Diagnostica Stago, Taverny, France). HDL, TC, triglyceride and LDL levels were measured using an automatic biochemistry analyzer (Hitachi 7600, Tokyo, Japan) and assayed by an enzymatic method according to the manufacturer’s instructions. Plasma hs-TnT levels were quantified utilizing an electrochemiluminescence immunoassay (IT3000, Roche, Switzerland). Given the heightened sensitivity, this assay is reported with units of picograms/milliliter (pg/mL). The determination of comorbidities relied on preadmission diagnoses or diagnoses established during hospitalization. To gather follow-up information, survival data from the Centers for Disease Control and Prevention were cross-referenced. Senior cardiologists provided oversight for quality control and conducted periodic data verification procedures.

### Outcome and definition

The study endpoints were cardiovascular and all-cause mortality. FHR was calculated as the plasma FIB concentration (g/L) divided by the plasma HDL-cholesterol level (mmol/L). Diagnoses of AMI, diabetes mellitus (DM), atrial fibrillation (AF) and hypertension were ascertained in accordance with the International Classification of Diseases, the tenth revision (ICD-10). Cardiovascular mortality was primarily identified by ICD-10 codes: I00–I99, Q20–Q28.

### Statistical analysis

The study stratified participants into four groups based on quartiles of baseline FHR values. Continuous variates were summarized as means (SD) or medians (IQR), while categorical variates were presented as counts and percentages. The variances among groups were evaluated utilizing one-way ANOVA, the Kruskal‒Wallis test and the Pearson chi-square test for continuous variates with normal or nonnormal distributions and categorical variates, respectively. The hazards of endpoints across different subsets were presented by Kaplan‒Meier methods. The correlation between baseline FHR levels and outcomes was illustrated by hazard ratio (HR, 95% CI) employed by Cox proportional hazard model. The established risk factors known to influence outcomes were selected as potential confounding covariates. Subsequently, multivariate stepwise Cox regression models were utilized to calculate the influencing variables of FHR (α_in_ = 0.05, α_out_ = 0.10). Three models were established sequentially: 1) without adjustment; 2) with adjustment for age and sex; 3) with further adjustment for covariates in Model 2, including smoking, monocyte count, TC, serum creatinine, LDL, triglycerides, use of antiplatelets, chronic kidney disease, congestive heart failure, stroke, hypertension, and DM. Restricted cubic spline (RCS) analyses were conducted to assess the potential nonlinear association between FHR and both cardiac and all-cause death, adjusting for the same covariates as mentioned above. Additionally, subgroup analyses were carried out, stratified by various demographic characteristics, comorbidities as well as laboratory examinations including age, sex, smoking status, DM, hypertension, stroke, LDL, TC and triglycerides. The study employed Youden’s index (sensitivity + specificity-1) and conducted an analysis of the area under the curve (AUC) for identifying the optimal cut-off value of mortality prediction. To evaluate whether incorporating the combination of FIB and HDL improved mortality prediction, the integrated discrimination improvement (IDI) and net reclassification improvement (NRI) were assessed. R software (version 4.2.1) was used for all analyses. In this analysis, if a two-tailed *P* value was below 0.05, statistical significance was present.

## Results

### Baseline characteristics

Figure 1 displays the study flowchart illustrating the process of selecting patients. In total, 13,221 patients were enrolled, and their baseline characteristics were comprehensively summarized in Table 1. Among the AMI population involved in the current study, the average age was 61.7 ± 12.0 years, with 81.9% of them being male. To ensure sufficient variability across the subgroups, the participants were divided into four subgroups, stratified by the quartiles of baseline FHR values which ranged from 0.002 to 0.998 and were distributed as follows in each subgroup: Quartile 1 (*N* = 3306), Quartile 2 (*N* = 3305), Quartile 3 (*N* = 3305), and Quartile 4 (*N* = 3305). The defined cutoff values for FHR were Q1 (< 3.11), Q2 (3.11–4.50), Q3 (4.50–6.35), and Q4 (> 6.35). Male and smoking participants were more prevalent in higher FHR quartiles compared with control group. Additionally, they exhibited a higher prevalence of coexisting conditions such as high blood pressure, congestive heart failure, DM, chronic kidney dysfunction, hyperlipidemia, and stroke. Conversely, the frequency of AF and anemia tended to be lower in the higher FHR quartiles. Among the laboratory parameters, individuals belonging to the upper quartiles of FHR exhibited higher levels of serum creatinine, monocytes and platelets. In contrast, patients in the higher levels of FHR in this study exhibited significantly lower TC, LDL, creatinine kinase MB, hemoglobin and albumin levels.

**Table 1:** Baseline demographics of entire population stratified by the level of FHR

**Baseline characteristics**	**Fibrinogen to HDL-cholesterol ratio**					
	**Overall**	**Q1 (< 3.11)**	**Q2 (3.11–4.50)**	**Q3 (4.50–6.35)**	**Q4 (> 6.35)**	***P*-value**
	***N* = 13,221**	***N* = 3306**	***N* = 3305**	***N* = 3305**	***N* = 3305**	
**Demographic characteristics**						
Age (years)	61.7 ± 12.0	62.9 ± 12.4	61.7 ± 12.2	61.5 ± 11.6	60.5 ± 11.7	< 0.001
Female	2393 (18.1)	739 (22.4)	661 (20.0)	603 (18.2)	390 (11.8)	< 0.001
Smoke	4970 (46.9)	1116 (43.8)	1200 (46.0)	1262 (46.9)	1392 (50.5)	< 0.001
**Medical history and clinical condition**						
CHF	4545 (34.4)	1055 (31.9)	1073 (32.5)	1102 (33.3)	1315 (39.8)	< 0.001
CKD	2969 (22.5)	589 (17.8)	650 (19.7)	814 (24.6)	916 (27.7)	< 0.001
DM	4307 (32.6)	761 (23.0)	1002 (30.3)	1206 (36.5)	1338 (40.5)	< 0.001
AF	532 (4.0)	180 (5.4)	114 (3.4)	122 (3.7)	116 (3.5)	< 0.001
Hypertension	6212 (47.0)	1405 (42.5)	1537 (46.5)	1623 (49.1)	1647 (49.8)	< 0.001
Hyperlipemia	9926 (75.1)	1912 (57.8)	2228 (67.4)	2650 (80.2)	3136 (94.9)	< 0.001
Anemia	469 (3.5)	143 (4.3)	101 (3.1)	111 (3.4)	114 (3.4)	0.035
Stroke	745 (5.6)	158 (4.8)	190 (5.7)	183 (5.5)	214 (6.5)	0.028
PCI	11,712 (88.6)	2803 (84.8)	2904 (87.9)	2966 (89.7)	3039 (92.0)	< 0.001
**Laboratory tests**						
Fibrinogen (g/L)	4.5 ± 1.6	2.9 ± 0.7	3.9 ± 0.8	4.9 ± 0.9	6.4 ± 1.3	< 0.001
APOA (g/L)	1.1 ± 0.3	1.3 ± 0.3	1.1 ± 0.2	1.0 ± 0.2	0.9 ± 0.2	< 0.001
APOB (g/L)	0.9 ± 0.3	1.0 ± 0.4	1.0 ± 0.3	0.9 ± 0.3	0.9 ± 0.2	< 0.001
TC (mmol/L)	4.7 ± 1.3	5.2 ± 1.3	4.8 ± 1.3	4.6 ± 1.2	4.2 ± 1.1	< 0.001
TG (mmol/L)	1.6 ± 1.2	1.5 ± 1.3	1.7 ± 1.2	1.7 ± 1.0	1.6 ± 1.0	< 0.001
LDL-C (mmol/L)	3.1 ± 1.1	3.5 ± 1.2	3.2 ± 1.1	3.0 ± 1.0	2.8 ± 0.9	< 0.001
HDL-C (mmol/L)	1.0 ± 0.3	1.3 ± 0.3	1.0 ± 0.2	0.9 ± 0.2	0.8 ± 0.2	< 0.001
Hemoglobin (g/L)	132.9 ± 18.3	136.4 ± 17.4	135.0 ± 17.9	132.1 ± 18.5	128.3 ± 18.3	< 0.001
Monocyte (10^9/L)	0.8 ± 0.4	0.7 ± 0.3	0.7 ± 0.4	0.8 ± 0.3	0.8 ± 0.4	< 0.001
SCr (umol/L)	1.1 ± 0.8	1.0 ± 0.5	1.1 ± 0.8	1.1 ± 0.8	1.3 ± 1.0	< 0.001
CKMB (U/L)	71.9 ± 171.5	149.1 ± 271.0	80.0 ± 155.2	37.1 ± 84.7	21.7 ± 57.1	< 0.001
WBC (10^9/L)	11.2 ± 83.1	12.3 ± 86.6	10.3 ± 14.4	12.3 ± 140.5	9.8 ± 11.5	0.489
PLT (10^9/L)	241.8 ± 80.3	225.2 ± 67.5	234.4 ± 73.8	244.2 ± 79.1	263.3 ± 93.5	< 0.001
ALB (g/L)	36.3 ± 5.2	39.6 ± 4.8	37.4 ± 4.6	35.4 ± 4.3	32.8 ± 4.6	< 0.001
**Medication during hospitalization**						
CCB	1167 (9.3)	230 (7.7)	296 (9.5)	333 (10.4)	308 (9.6)	0.003
Statins	11,959 (95.7)	2857 (96.2)	2989 (95.7)	3036 (95.0)	3077 (95.8)	0.097
β-blockers	10,064 (80.5)	2210 (74.4)	2533 (81.1)	2646 (82.8)	2675 (83.3)	< 0.001
ACEI/ARB	8553 (68.4)	1810 (61.0)	2173 (69.6)	2288 (71.6)	2282 (71.1)	< 0.001
Antiplatelets	12,365 (98.9)	2926 (98.6)	3087 (98.9)	3160 (98.8)	3192 (99.4)	0.011

*Abbreviation*: *CHF* congestive heart failure, *CKD* chronic kidney disease, *DM* diabetes mellitus, *AF* atrial fibrillation, *PCI* percutaneous interventions, *APOA* apolipoprotein A-I, *APOB* apolipoprotein B, *TC* total cholesterol, *TG* triglyceride, *LDL-C* low density lipoprotein cholesterol, *HDL-C* high density lipoprotein cholesterol, *SCr* serum creatinine, *CKMB* creatine kinase MB, *WBC* white blood cell, *PLT* platelet, *ALB* albumin, *CCB* calcium channel blocker, *ACEI* angiotensin converting enzyme inhibitor, *ARB* angiotensin receptor blocker

### Predictive value of FHR on all-cause mortality

**Fig. 2:**
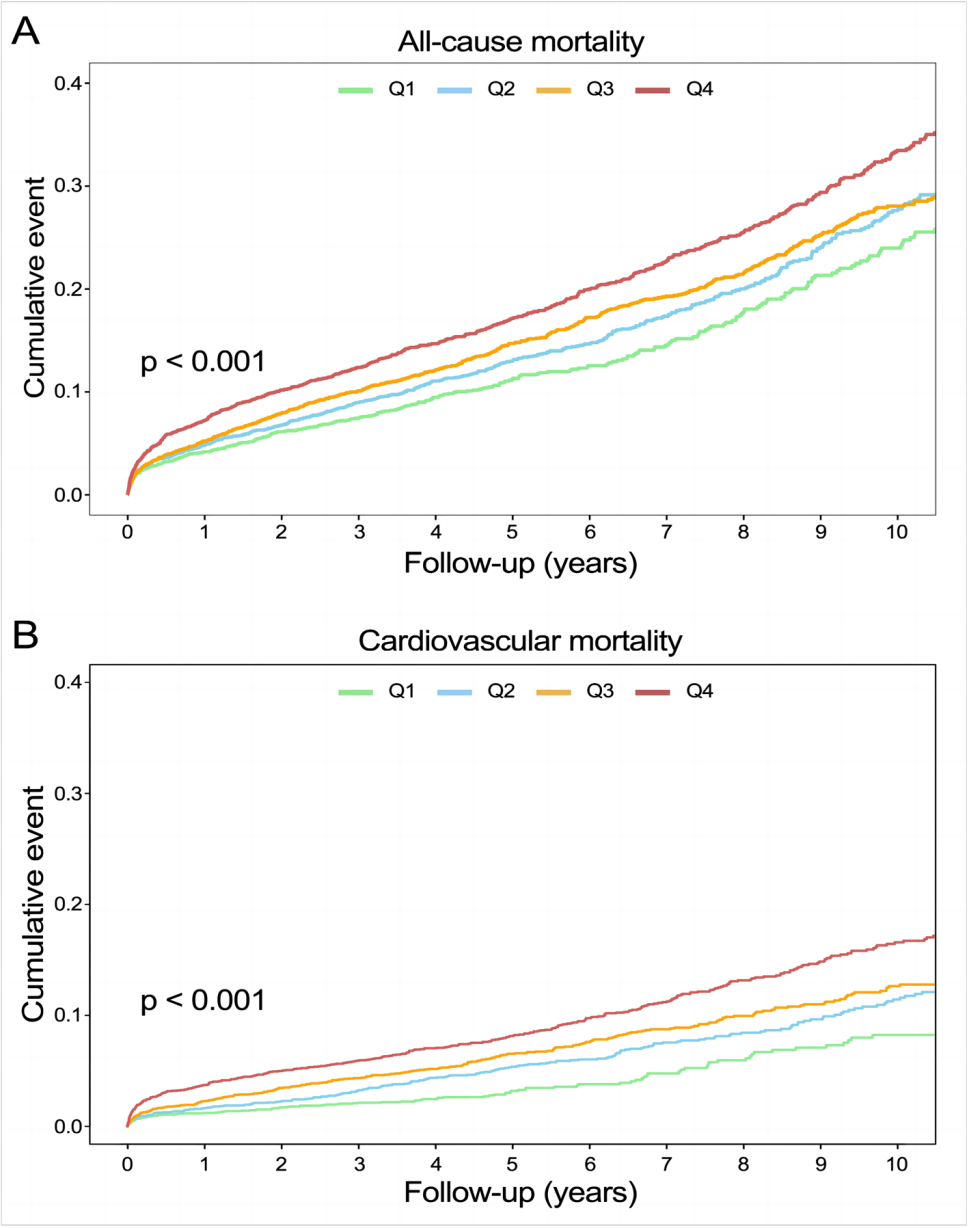
Kaplan–Meier analysis for all-cause (**A**) and cardiovascular mortality (**B**) according to different FHR levels

**Fig. 3:**
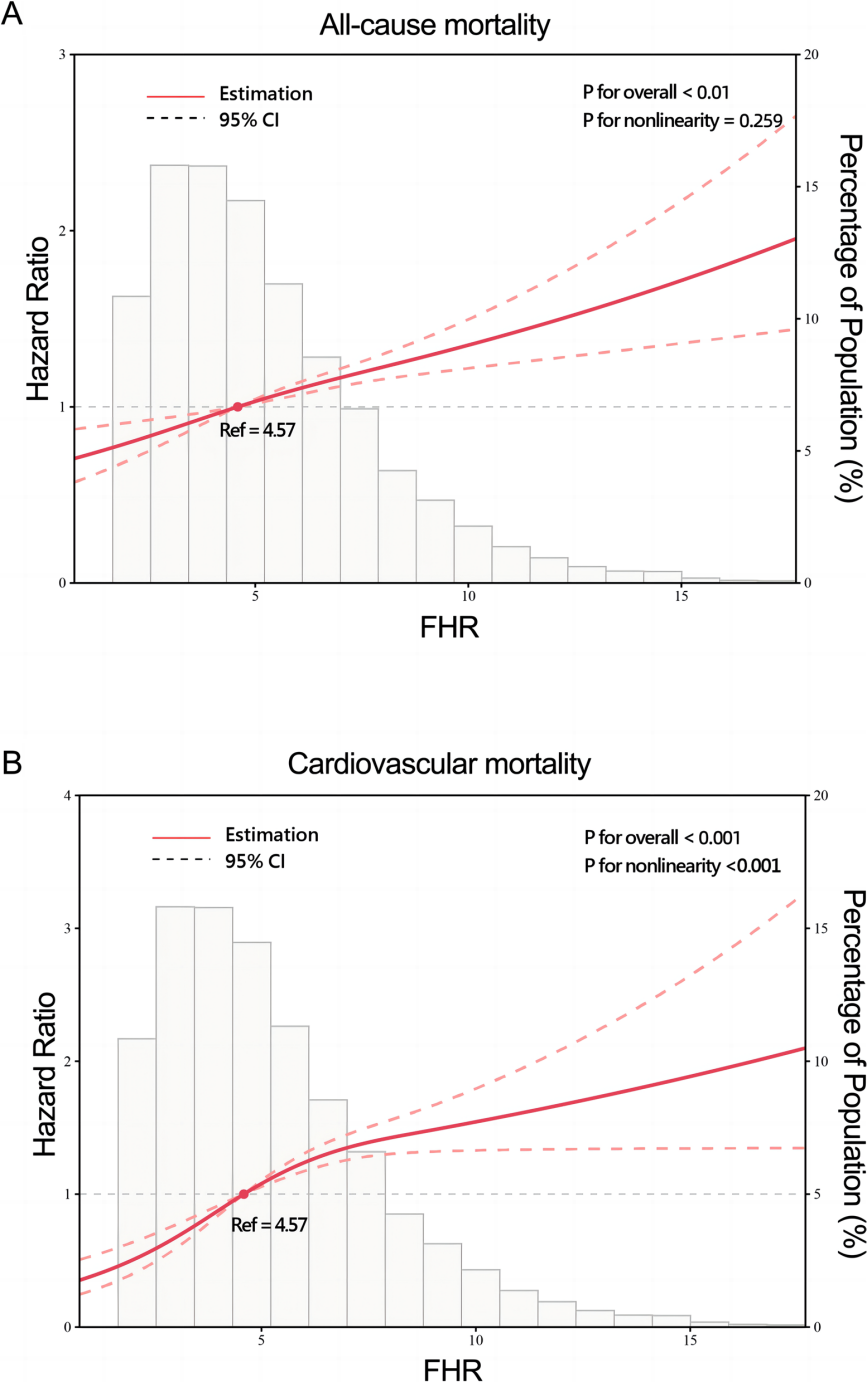
Restricted cubic splines for the relationship between FHR and all-cause (**A**) and cardiovascular mortality. **B** Adjusted for age, sex, smoking, LDL, triglyceride, total cholesterol, serum creatinine, monocyte count, use of antiplatelets, hypertension, chronic kidney disease, congestive heart failure, diabetes mellitus and stroke

A total of 2,309 (17.5%) patients encountered mortality throughout the 10-year follow-up. The occurrence of all-cause mortality across FHR quartiles was as follows: Q1-11.6% (384/3306), Q2-16.5% (544/3305), Q3-18.8% (620/3305), and Q4-23.0% (761/3305). Kaplan‒Meier analysis curves demonstrated progressively adverse outcomes with elevated FHR levels (*P* < 0.001, Fig. 2A). Furthermore, data for the enrolled patients were subjected to Cox regression analysis to assess the prognostic relevance of various FHR values (Table 2). The unadjusted model showed that individuals in higher quartiles exhibited a greater likelihood of mortality due to all causes than individuals in the first quartile (the reference group) (HR 1.18, 95% CI 1.04–1.35, *P* = 0.013; HR 1.28, 95% CI 1.23–1.45, *P* < 0.001; HR 1.55, 95% CI 1.37–1.76, *P* < 0.001; respectively). These findings remained statistically significant even after comprehensive adjustment for covariates in the fully adjusted analysis, including age, sex, smoking, LDL, triglyceride, TC, serum creatinine, monocyte count, use of antiplatelets, hypertension, chronic kidney disease, congestive heart failure, DM and stroke (Table 2; HR 1.16, 95% CI 0.98–1.37, *P* = 0.09; HR 1.25, 95% CI 1.06–1.47, *P* = 0.008; HR 1.48, 95% CI 1.26–1.75, *P* < 0.001; respectively). Moreover, RCS models revealed that FHR was linearly correlated with all-cause mortality, as evidenced by both the unadjusted and adjusted models (Fig. 3A & Figure S1: *P* value for nonlinearity > 0.05).

**Table 2:** Predictive value of the FHR level for all-cause and cardiovascular mortality

**FHR**	**Model1**		**Model2**		**Model3**	
	**HR (95%CI)**	***P*-value**	**HR (95%CI)**	***P*-value**	**HR (95%CI)**	***P*-value**
**All-cause mortality**						
Quartile 1	Ref		Ref		Ref	
Quartile 2	1.18 (1.04–1.35)	0.013	1.24 (1.09–1.41)	0.001	1.16 (0.98–1.37)	0.090
Quartile 3	1.28 (1.23–1.45)	< 0.001	1.35 (1.19–1.54)	< 0.001	1.25 (1.06–1.47)	0.008
Quartile 4	1.55 (1.37–1.76)	< 0.001	1.74 (1.54–1.97)	< 0.001	1.48 (1.26–1.75)	< 0.001
**Cardiovascular mortality**						
Quartile 1	Ref		Ref		Ref	
Quartile 2	1.55 (1.24–1.94)	< 0.001	1.66 (1.33–2.07)	< 0.001	1.84 (1.35–2.50)	< 0.001
Quartile 3	1.82 (1.47–2.25)	< 0.001	1.97 (1.59–2.44)	< 0.001	2.15 (1.59–2.91)	< 0.001
Quartile 4	2.45 (1.99–3.01)	< 0.001	2.88 (2.34–3.54)	< 0.001	2.70 (1.99–3.65)	< 0.001

Model 1: Not adjusted; Model 2: adjusted for age and sex; Model 3: adjusted for age, sex, smoking, LDL, triglyceride, total cholesterol, serum creatinine, monocyte count, use of antiplatelets, hypertension, chronic kidney disease, congestive heart failure, diabetes mellitus and stroke

### Association of FHR with cardiovascular mortality

Over a median follow-up of 4.66 (2.48–7.48) years, 2309 patients experienced all-cause mortality, with 1007 deaths attributed to cardiovascular causes. The incidence of cardiovascular mortality across the FHR quartiles was presented as follows: Q1-3.6% (120/3306), Q2-6.8% (226/3305), Q3-8.5% (280/3305), and Q4-11.5% (381/3305). The Kaplan‒Meier plot illustrated a statistically significant association between elevated FHR values and diminished survival in AMI patients (Fig. 2B; *P* < 0.001). Based on the adjustment for potential confounders (Table 2), a higher FHR was consistently correlated with a higher likelihood of mortality due to CVD. The HRs and 95% CIs were as follows: Q2 – HR 1.84, 95% CI 1.35–2.50, *P* < 0.001; Q3 – HR 2.15, 95% CI 1.59–2.91, *P* < 0.001; Q4 – HR 2.70, 95% CI 1.99–3.65, *P* < 0.001. To further explore the correlation between FHR and cardiac mortality, RCS models were utilized, revealing a significant nonlinear association between FHR and cardiovascular death in patients diagnosed with AMI (*P* for nonlinearity < 0.001) (Fig. 3B).

### Subgroup analysis

**Fig. 4:**
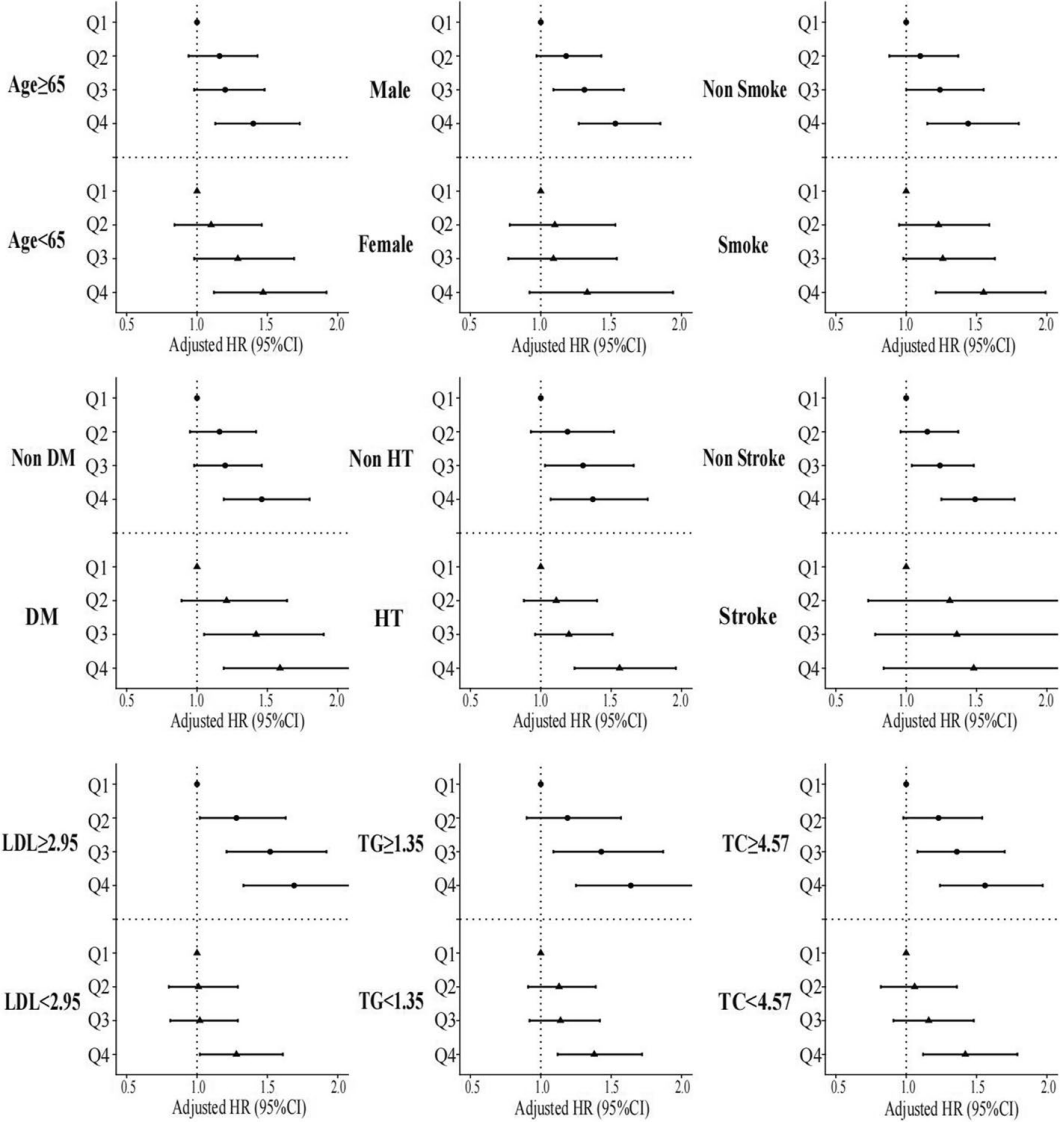
Forest plot of all-cause mortality according to different subgroups.  Adjusted for age, sex, smoking, LDL, triglyceride, total cholesterol, serum creatinine, monocyte count, use of antiplatelets, hypertension, chronic kidney disease, congestive heart failure, diabetes mellitus and stroke

To assess potential interactions between the four FHR subsets and various covariates (age, sex, smoking status, DM, hypertension, stroke, LDL, triglycerides and total cholesterol) in relation to all-cause mortality, post-hoc subgroup analysis was performed (Fig. 4). Interestingly, patients in Q2, Q3, and Q4 exhibited consistent characteristics in certain subsets (male, non-smoker, DM, non-hypertension, non-stroke, LDL ≥ 2.95 mmol/L, triglyceride ≥ 1.35 mmol/L and total cholesterol ≥ 4.57 mmol/L), compared to ones in quartile 1 (*P* for interaction > 0.05).

### Association of FHR with all-cause and cardiovascular mortality

The predictive value of FHR, FIB, HDL, hs-TnT as well as the combination of FHR and hs-TnT for cardiovascular mortality risk assessment among AMI patients was conducted through Receiver operating characteristic curve analysis (Figure S2-5). For FHR, the AUC was 0.624 (95% CI 0.607–0.642) and the optimal cut-off value was 4.38, with sensitivity and specificity recorded at 68% and 50%, respectively (Figure S3). As depicted in Figure S2, the cut-off value for hs-TnT was 1208.50 pg/mL, with a sensitivity of 64% and specificity of 51%. Notably, in both scenarios, the AUC for FHR, at 0.624, exceeded that of hs-TnT, which measured 0.592 (95% CI 0.564–0.621), as well as FIB (AUC 0.613, 95% CI 0.596–0.630) and HDL (AUC 0.558, 95% CI 0.539–0.576) (Figure S5). Additionally, the combination of FHR and hs-TnT for predicting cardiovascular mortality achieved the utmost AUC at 0.627 (95% CI 0.598–0.656) (Figure S4). Similar results were observed in predicting all-cause mortality. Further, the improved predictive capacity of the combination of FIB and HDL was assessed by IDI and NRI as shown in Table S1. Obviously, both IDI and NRI indicated that FHR led to a slight but significant improvement in all-cause and cardiovascular mortality prediction.

## Discussion

This retrospective real-world study was conducted on a substantial cohort from China, with a period of 10-year follow-up. The current study investigated the joint effect of plasma HDL and FIB levels in predicting adverse outcomes among AMI patients. The findings demonstrated that the integrated categorization of HDL and FIB enhanced the predictive value for adverse outcomes, incrementally increasing the likelihood of death owing to cardiac and all-cause events. Multivariate Cox analysis also indicated a higher risk of mortality among individuals within the highest FHR quartile than others. Even after adjusting for confounding variables, patients in Q4 exhibited a 48% increased risk of all-cause death as well as a 1.7-fold increase in cardiovascular mortality in comparison to ones in reference group. Additionally, the RCS curve demonstrated elevated FHR was linearly correlated to all-cause mortality. Furthermore, receiver operating characteristic curve analysis indicated that FHR, with an AUC of 0.624, outperformed hs-TnT (AUC: 0.592) individually, and the combined use of both biomarkers yielded the highest AUC (0.627) in predicting cardiac and all-cause mortality. Moreover, the IDI and NRI analysis for FHR in predicting mortality exhibited significant improvement compared to those for FIB and HDL. These results highlight the potential of combining FIB, an indicator of inflammation and coagulation state, with HDL, a complex circulating lipoprotein, which could enhance the predictive capacity in subsequent risk stratification of AMI patients.

FIB, a liver-synthesized serum glycoprotein, is of great importance in both the inflammatory and coagulation cascades, making it a key factor in the formation and progression of coronary atherosclerosis [7]. Several studies have revealed its significance in various aspects of CVD previously [21, 22]. For instance, a case–control study [23] revealed significantly elevated FIB levels 3 to 6 months after hospitalization for CVD in comparison to healthy controls. Individuals in the top quartile of FIB exhibited an odds ratio of 6.0 (95% CI 3.5–10.4) in comparison to ones in the lowest group after age adjustment. Similarly, in a prospective investigation [24], FIB levels were assessed 6 months prior to study entry, and differences in FIB levels were observed between survivors and those who died. Moreover, another study explored the association of FIB with cardiac adverse events following AMI, reporting significantly higher FIB levels in individuals with a prior history of AMI or peripheral artery disease in comparison to those without prevalent CVD [25]. In addition, FIB levels were found to correlate with early alterations in the carotid artery due to atherosclerosis, even among individuals with minimal CVD risk [26]. On the other hand, HDL has exhibited numerous protective benefits in cardiac disease mainly attributed to its ability to exert cholesterol efflux, anti-inflammatory and anti-oxidant [27] properties on endothelial cells and macrophages, etc. [28]. Cockerill et al. illustrated that physiological concentrations of HDLs isolated from healthy donors decreased the expression of endothelial adhesion molecules induced by cytokines [29]. Additionally, within the coagulation cascade, HDL plays a multifaceted regulatory function, as indicated by its positive correlation with anticoagulant responses and its ability to counteract the procoagulant characteristics of anionic phospholipids [15]. Considering that plasma FIB and HDL hold significance in coagulation and inflammatory alterations and are closely linked to cardiovascular incidents, further investigations were essential to assess whether their interplay, like FHR, might assist in identifying individuals at high risk within the CVD population.

A prior study by Ma et al. [19] demonstrated a correlation between concurrent increases in FIB and declines in HDL levels and an increased risk of recurrent cerebral thrombosis, whereas the separate evaluation of FIB and HDL levels did not yield significant results. According to a study by Kowalski et al. [30], the co-occurrence of elevated D-dimer, a breakdown product of FIB, and lower HDL levels appeared to contribute to the progression of acute pulmonary emboli. Moreover, other studies indicated a strong association between simultaneous elevation of FIB and reduction in HDL levels with recurrent CVT in previously diagnosed CVT patients, as well as the onset of CVD in the DM cohort [20]. In addition, Sung et al. observed a significant higher FIB and lower HDL levels in patients enrolled from the outpatient department who experienced major adverse cardiovascular events than in those who did not. Notably, an inverse relationship was observed between FIB and HDL levels, which implies an interaction between FIB and HDL may exacerbate atherosclerosis and thrombosis [31]. Moreover, another study revealed a relationship between FHR and idiopathic sudden sensorineural hearing loss, a condition with a higher prevalence among individuals with underlying inflammatory and systemic vascular diseases such as atherosclerosis and diabetes mellitus [32]. Treatment outcomes were classified into four groups in accordance with the degree of hearing recovery, and FHR was found to be significantly lower in groups associated with better outcomes. This study suggested FHR might be a valuable prognostic indicator for hearing recovery among those patients [33].

In various cardiological studies, the monocyte/HDL ratio (MHR) and neutrophil/HDL ratio (NHR) have been explored as useful inflammatory biomarkers for predicting adverse cardiac outcomes. Lütfü Aşkın et al. evaluated MHR in 99 consecutive STEMI patients, classifying them into two subsets based on the median of QRS score. The results indicated a correlation between elevated MHR and a higher QRS score, suggesting its potential as an independent predictor for high QRS scores in STEMI patients [34]. In another research conducted by Huang et al., NHR was evaluated among 528 elderly AMI patients. They found NHR was linked to long-term mortality and recurrent MI, which might serve as a predictor for worse clinical outcomes of elderly AMI patients [35]. In addition, other studies have illustrated the potential role of von Willebrand factor (vWF) as a pro-atherogenic biomarker predicting adverse cardiac outcomes [36]. For instance, Mario et al. have shown that shear-induced platelet aggregation correlated with enhanced vWF concentration among AMI patients [37]. Rutten et al. observed a substantial increase in active vWF levels among individuals experiencing ST-elevation MI for the first time compared to controls (*P* < 0.0001), emphasizing the central role of vWF in the progression of thrombosis [38]. Moreover, Sergio et al. investigated the prognostic role of hemoglobin decline among patients with acute coronary syndrome (ACS). Their study involved 7,781 invasively managed ACS patients, categorized based on in-hospital hemoglobin decline, and further subdivided according to the presence of adjudicated in-hospital bleeding. The findings revealed a decline in hemoglobin of ≥ 3 g/dL during hospitalization, irrespective of overt bleeding, was independently linked to a higher risk of 1-year mortality [39].

In our study, we compared FHR with hs-TnT for mortality prediction. Cardiac troponin T, a biomarker widely recommended for diagnosing AMI [40], has been found independently associated with adverse outcomes following acute coronary syndrome [41]. James et al. assessed the role of hs-TnT in 3,546 individuals from the Dallas Heart Study, revealing that baseline hs-TnT concentrations were linked to the presence of structural heart disease and subsequent risk of total mortality [42].

The current study is the first to evaluate the concurrent presence of both parameters in individuals with AMI using FHR. FIB and HDL levels have previously been assessed separately, showing implications in inflammatory and atherothrombotic processes [43]. The research revealed a statistically significant association between elevated FHR values and worse outcomes among AMI patients. These results highlight that the joint effect of HDL and FIB could augment mortality prediction in AMI population. This enhanced predictive value could facilitate future risk stratification for the AMI population. Thus, incorporating these two fundamental markers in clinical practice could be advantageous.

### Study strengths and limitations

The current study illustrated elevated FHR values were strongly correlated with enhanced all-cause as well as cardiac mortality among AMI patients, for the first time. Considering the clinical burden of complications associated with CVD, the assessment of FHR holds the potential to serve as a powerful indicator of long-term mortality among AMI patients. However, this research exists a few limitations. Firstly, the predominance of male participants in this study (4.5:1) may introduce bias and limit the generalizability of our findings. It is essential to acknowledge that this study was conducted on a population from southern China hospitalized with AMI, which may not fully represent the broader Chinese population. Additionally, the inverse association between FHR and TC/LDL-cholesterol may indicate different clinical phases and baseline status of inflammation or malnutrition among this population, which might potentially bias the study results. Further investigations are requested to assess if those outcomes could be extrapolated to other populations. Secondly, the measurements of HDL and FIB concentrations were only conducted at baseline, but changes in these biomarkers during the follow-up period may also hold clinical significance. Thirdly, despite the comprehensive adjustment for potential risk factors in the analysis, certain variables could not be measured or acquired, potentially resulting in residual confounding which might be unavoidable. Fourthly, the CIN-II cohort lacks data regarding GRACE scores, a risk assessment instrument utilized in patients with ACS as well as left ventricular ejection fractions. Consequently, we opted to employ hs-TnT as an alternative to predict long-term mortality among AMI patients for comparison. Although there were studies demonstrating association between hs-TnT and adverse cardiovascular outcomes, it is better representing acute mortality after the ischemic event. Lastly, as this was an observational study, the underlying mechanisms behind the association require further investigation.

## Conclusion

This real-world cohort study revealed that higher FHR values were independently related to adverse clinical outcomes among AMI patients, suggesting FHR holds potential as a prognostic indicator to identify individuals at higher risk of mortality in the context of AMI. Since FHR can be easily and inexpensively measured, it might contribute to improved clinical decision-making and patient management for AMI patients.

### Supplementary Information

**Supplementary Material 1.**

## Data Availability

No datasets were generated or analysed during the current study.

## Abbreviations

AMI: Acute myocardial infarction
HDL: High density lipoprotein
FHR: Fibrinogen to HDL-cholesterol ratio
FIB: Fibrinogen
CVD: Cardiovascular disease
AF: Atrial fibrillation
CVT: Cerebral venous thrombosis
DM: Diabetes mellitus
LDL: Low density lipoprotein
TC: Total cholesterol
hs-TnT: High-sensitivity troponin T
vWF: Von Willebrand factor
ACS: Acute coronary syndrome
AUC: Area under the curve
RCS: Restricted cubic spline
IDI: Integrated discrimination improvement
NRI: Net reclassification improvement
