# Supplementary material for: Fibrinogen to HDL-Cholesterol ratio as a predictor of mortality risk in patients with acute myocardial infarction
Source: Lipids Health Dis. 2024 Mar 25;23:86. doi: 10.1186/s12944-024-02071-7 (PMC10962130; doi:10.1186/s12944-024-02071-7)

**Supplementary Table & Figure legend**

**Table S1.** Evaluation of enhanced predictive ability for endpoints using IDI and NRI.

**Figure S1**. Restricted cubic splines of FHR levels in relation to crude HR (A) and adjusted HR (B & C) for the risk of all-cause mortality.

A: unadjusted; B: adjusted for age and sex; C: adjusted for age, sex, smoke, LDL, triglyceride, total cholesterol, serum creatinine, monocyte count, use of antiplatelets, hypertension, chronic kidney disease, congestive heart failure, diabetes mellitus and stroke.

**Figure S2.** Receiver operating characteristic curve of the prediction for 10-year cardiovascular mortality based on hs-TnT.

**Figure S3.** Receiver operating characteristic curve analysis for predicting 10-year cardiovascular mortality based on FHR levels.

**Figure S4.** Receiver operating characteristic curve of the prediction for 10-year cardiovascular mortality based on the combination of FHR and hs-TnT.

**Figure S5.** Receiver operating characteristic curve of FHR, FIB, HDL and hs-TnT for predicting 10-year cardiovascular mortality.

**Table S1.** **Evaluation of enhanced predictive ability for endpoints using IDI and NRI.**

| **Comparison** | **Item** | **Estimation** | **95%CI** | ***P*-value** |
| --- | --- | --- | --- | --- |
| **All-cause mortality** | | | | |
| FHR vs FIB | IDI | 0.002 | 0.000-0.003 | 0.024 |
|  | NRI | 0.013 | 0.013-0.076 | 0.016 |
| FHR vs HDLC | IDI | 0.004 | 0.001-0.006 | <0.001 |
|  | NRI | 0.041 | 0.014-0.069 | <0.001 |
| **Cardiovascular mortality** | | | | |
| FHR vs FIB | IDI | 0.006 | 0.002-0.009 | 0.008 |
|  | NRI | 0.125 | 0.004-0.144 | 0.008 |
| FHR vs HDLC | IDI | 0.013 | 0.005-0.019 | <0.001 |
|  | NRI | 0.124 | 0.000-0.147 | 0.004 |

Abbreviation: FHR= fibrinogen to HDL-cholesterol ratio; FIB= fibrinogen; HDLC= HDL-cholesterol ratio; IDI=integrated discrimination improvement; NRI= net

reclassification improvement.


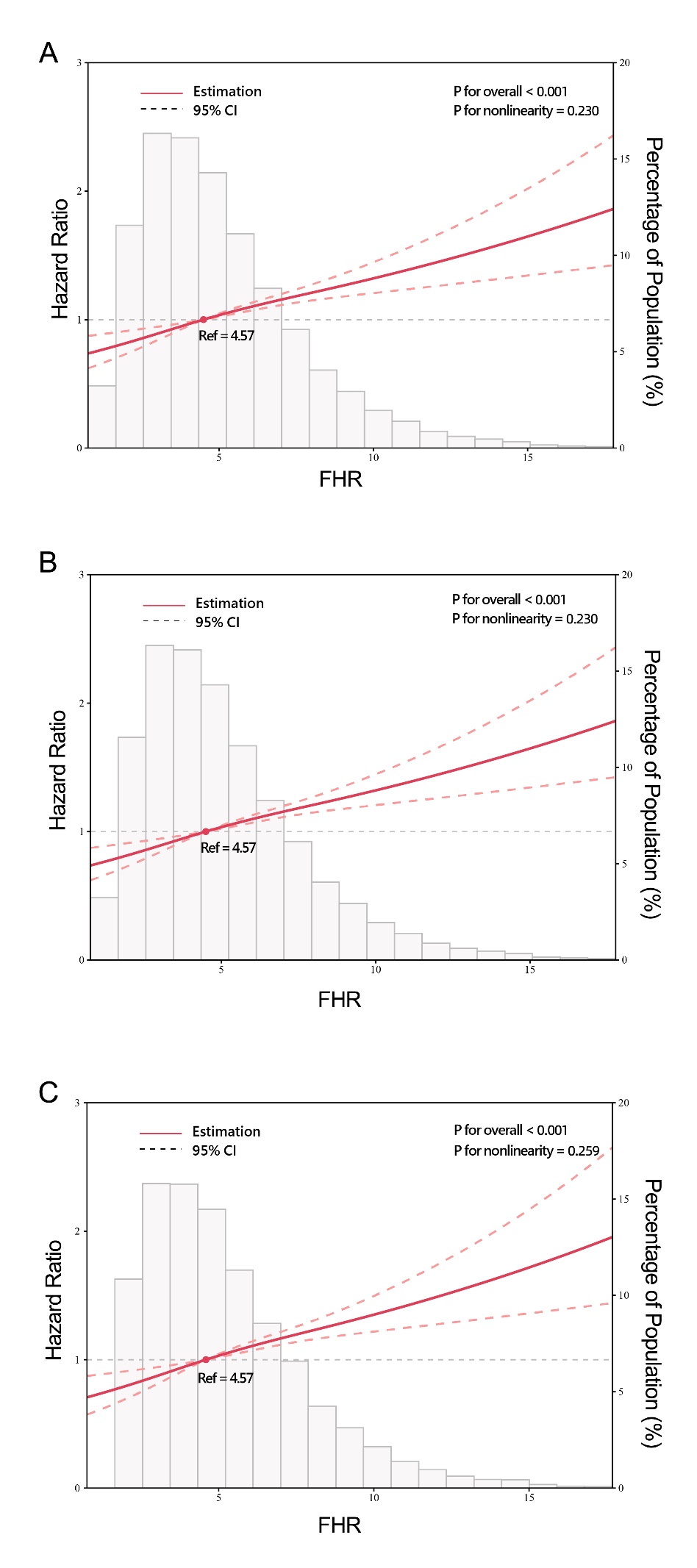


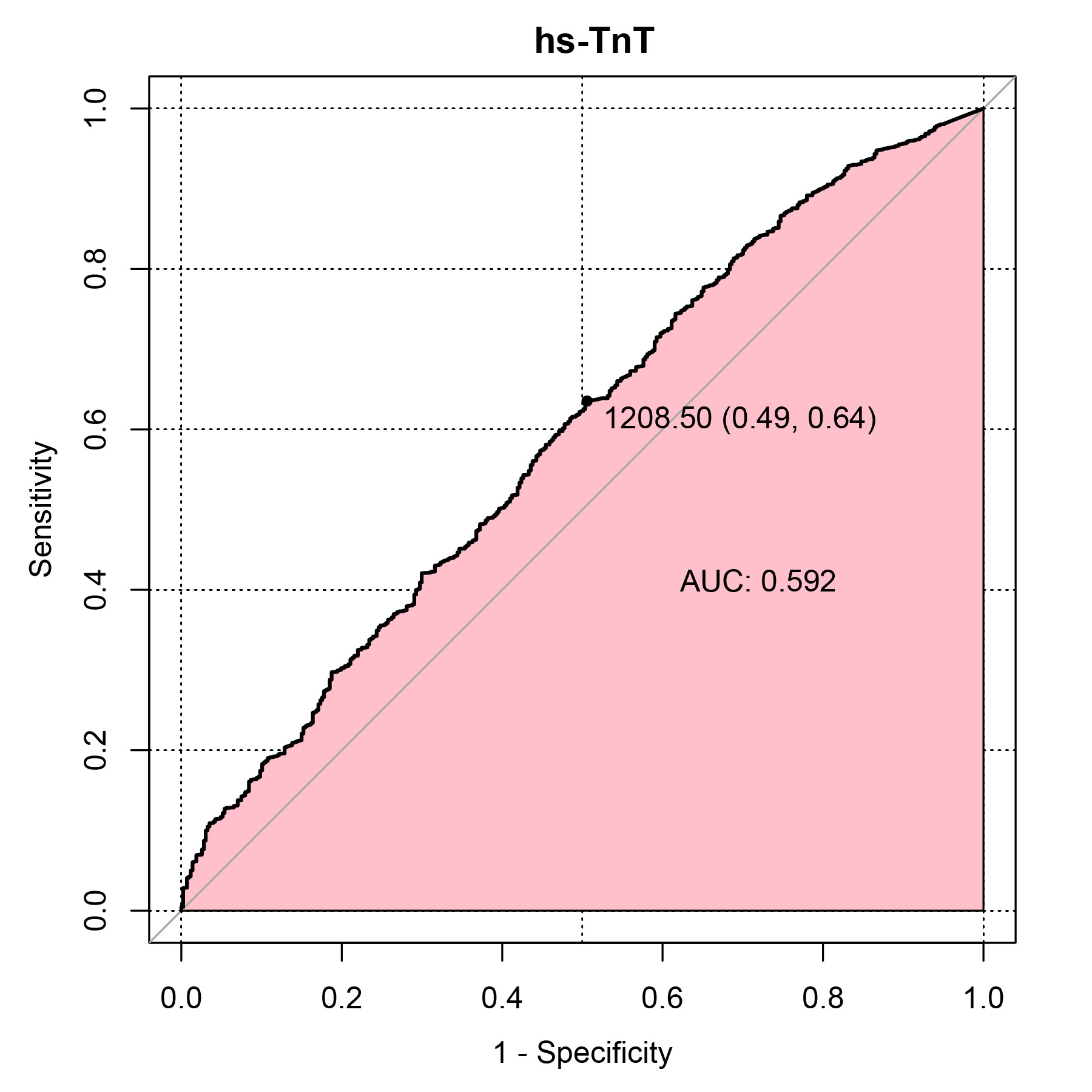


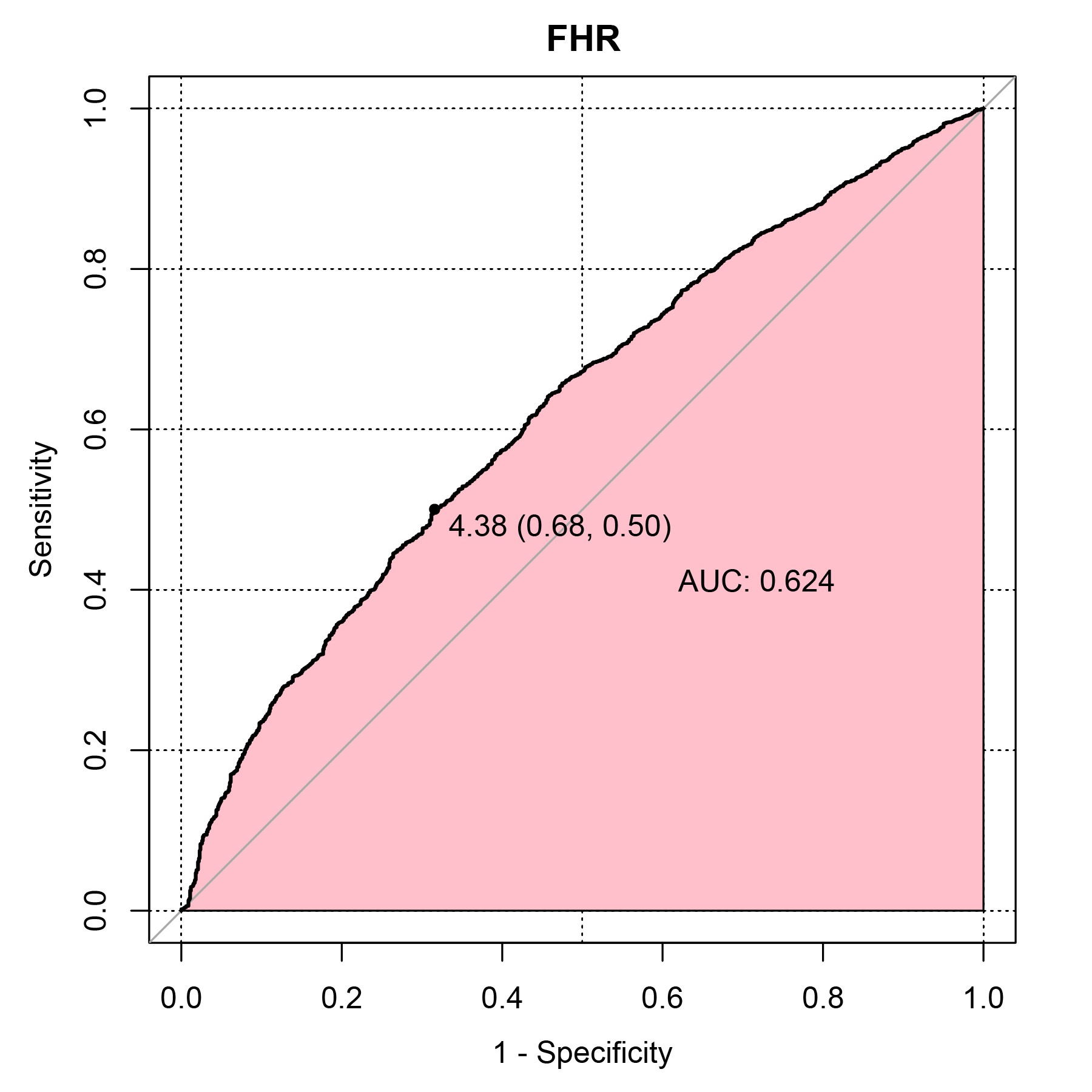


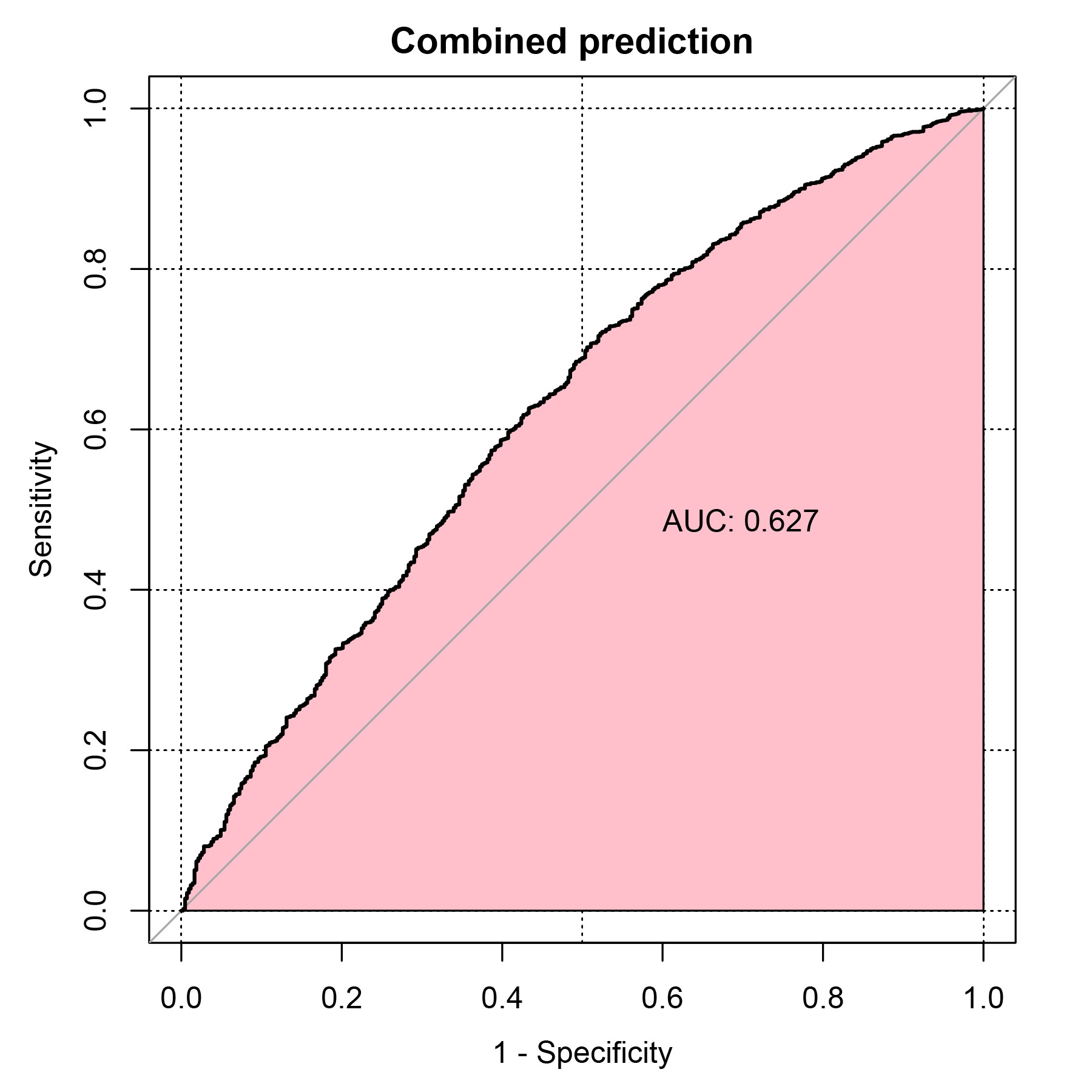


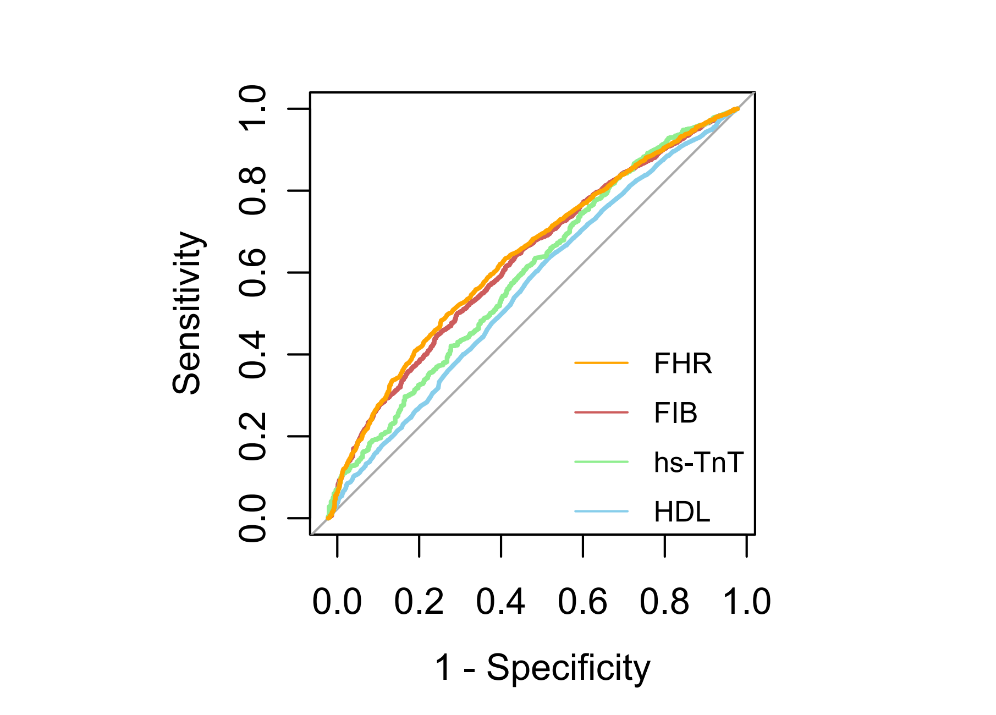

Supplement: Supplementary file 1 — Supplementary Material 1. [file 12944_2024_2071_MOESM1_ESM.docx]
